# The role of quantitative ultrasound in diagnosing severe bone metabolic diseases in newborns

**DOI:** 10.3389/fped.2023.1109553

**Published:** 2023-04-11

**Authors:** Sandra Cerar, Darja Paro-Panjan, Aneta Soltirovska-Šalamon

**Affiliations:** ^1^ Division of Paediatrics, Department of Neonatology, University Medical Centre Ljubljana, Ljubljana, Slovenia; ^2^ Department of Paediatrics, Faculty of Medicine, University of Ljubljana, Ljubljana, Slovenia

**Keywords:** bone mineral density, quantitative ultrasound, newborn, metabolic bone disease, osteogenesis imperfecta, hypophosphatasia

## 1. Introduction

Metabolic bone diseases (MBD) are heterogeneous in aetiology, onset and severity, and are commonly associated with an increased risk of fractures in childhood. The diagnostic process is often challenging despite a scrupulous analysis of skeletal radiographs and laboratory tests (1, 2).

Assessment of bone mineral density (BMD) can be performed by multiple imaging methods, but currently none of them provides a full evaluation of bone health in children and adults (3). To date, dual-energy X-ray absorptiometry (DXA) is the reference standard for assessing BMD in children and adults, as recommended by the International Society for Clinical Densitometry (ISCD) (4). However, in the neonatal period the method has several limitations, including radiation and the lack of reference data. Therefore, Quantitative ultrasound (QUS) may be advantageous in assessing bone health in infants: it is easy to use, radiation-free technique with high reproducibility and can be used in correlation with DXA (1, 3, 5).

The aim of this case series is to describe the role of QUS in the neonatal period in detecting low BMD in 3 neonates with severe MBD.

## 2. Cases presentation

The first case was a male infant with hypophosphatasia (HPP). A full-term male, born after an uneventful pregnancy and delivery, with appropriate measurements for gestational age, and Apgar score 8/9/9 presented in the first week of life due to failure to thrive and apneas. In a few days episodes of tonic convulsions occurred. Clinical examination revealed severe generalized hypotonia, slightly shorter upper extremities, and widely opened fontanels and sagittal sutures. Cranial ultrasound, brain magnetic resonance imaging, a hearing test and ophthalmological examination were normal, and electroencephalogram showed normal activity. QUS revealed osteoporosis (SOS 2890 m/s, Z score: >−2.5). Laboratory tests were normal, but the level of alkaline phosphatase was unmeasurably low, and the level of its substrate—phosphoethanolamine—was increased. HPP was then confirmed by genetic analysis (in the ALPL gene: a missense mutation in exon 12 (*c.1400 T > C, p.Met467Thr*) and a novel mutation, heterozygous frameshift deletion of two nucleotides in exon 10 (c.1114_1115delCT, p.Leu372Aspfs*32)). Seizures did not re-occur with maintenance therapy with pyridoxine. Weight gain was inadequate, chest deformities became apparent, and severe hypotonia persisted. Before receiving treatment with enzymatic replacement therapy, he died in early infancy due to acute respiratory failure.

The 2nd and 3rd cases were male infants with osteogenesis imperfecta (OI). Their common antenatal history included detection of shorter bones of the lower extremities (<1. percentile**)**, followed by amniocentesis; karyotype and molecular karyotype were normal; achondroplasia (*FGFR3* gene) was excluded. Both were vaginally delivered with high Apgar scores. The 2nd case was born prematurely (36 weeks of gestation) as symmetrically small for gestational age. He presented with bluish discoloration of the sclera, wide sutures, thin skull bones, deformed long bones of the lower extremities, hypermobile joints, and central hypotonia. QUS showed osteopenia (SOS 2830 m/s, Z score −1.8). Except for the atrial septal defect, no other congenital abnormalities were found. Two fractures (femur and humerus) were identified by x-ray at the age of 16 days. OI was confirmed by genetic analysis (gene mutation *COL1A2*). Bisphosphonate treatment was introduced. The BMD, followed by QUS in the first year, was in the range of osteopenia (Z score–1.8), and also at the age of 15 months (Z score–2.4), measured by DXA. In the course of time osteoporosis evolved (Z score–2.5). The gradual deformity of the chest led to respiratory insufficiency; night-time non-invasive respiratory support was required until the age of 4 years. By the age of 8 years he had suffered 8 fractures. He is of short stature and his BMD remains in the range of osteoporosis.

The 3rd case was born at term with appropriate anthropometric birth measurements. He presented with widely opened cranial sutures, bowed and shortened lower extremities, and central hypotonia. QUS revealed osteoporosis (SOS 2714 m/s, Z score −3.1). Hypoplasia of the optic nerves was found on ophthalmological assessment. On the 17th day X-ray revealed bilateral femur fractures and additional fractures of the humerus and radius, but no other fractures were detected by total body MRI. Genetic analysis revealed osteogenesis imperfecta (gene mutation *COL1A2*). Bisphosphonate treatment was introduced at the age of 5 weeks. Follow-up measurements of BMD with QUS remained in the range of osteoporosis. In both cases bone metabolism laboratory results were normal. Due to inadequate callus formation immobilisation was prolonged to a total of 4 weeks. Calcium, phosphate and D vitamin have been supplemented since infancy.

## 3. Discussion

In this case series, QUS has been shown to be useful in the identification of neonates with MBD with a genetic background. Low BMD assessed by QUS, in association with the clinical presentation, directed further investigations toward a prompt final diagnosis. Additionally, the utility of QUS has been demonstrated during the follow up of BMD and treatment response.

The most challenging was the 1st case: the neonate presented with relatively unspecific clinical signs-failure to thrive and convulsions. Although severe neurological impairment, including seizures, was present, skeletal involvement was suspected due to the slightly shorter upper extremities, and widely opened fontanels and sagittal sutures. Identifying severe osteoporosis by QUS, a low alkaline phosphatase level, in combination with pyridoxine-responsive seizures, directed further investigations (assessing the levels of tissue non-specific alkaline phosphatase substrates—phosphoethanolamine, which is a specific diagnostic marker) toward HPP. MBD might be suspected even in newborns with subtle abnormal clinical and neurological signs. Contrary to the 1st case, MBD was strongly suspected in both the 2nd and 3rd cases. Considering the clinical signs and identifying low BMD by QUS confirmed the suspicion of MBD in a few days after birth. The 2nd neonate was born small for gestational age with bluish discoloration of the sclera, wide sutures, thin skull bones, deformed long bones of the lower extremities, and hypermobile joints. Measurements of BMD by QUS were in the range of osteopenia, and were comparable to the follow-up measurements of BMD with DXA at the age of 15 months. Subsequently, according to the DXA measurement, osteoporosis evolved, and despite treatment with bisphosphonate and mineral supplementation, skeletal deformities and several fractures occurred.

Furthermore, the 3rd case was even more interesting. In accordance with the initial clinical presentation (widely opened sutures, curved and shortened lower extremities), MBD was also suspected and BMD, measured by QUS, was found to be in the range of osteoporosis two weeks prior to the appearance of the first fractures. This finding led us to conduct prompt targeted genetic testing. Although BMD is significantly reduced in OI, quantitative measurement of BMD is not an obligatory criteria for diagnosis (6). Additionally, this information was helpful for fracture awareness and recognition before genetic confirmation of the disease. Nonetheless, as the osteoporosis was consistent with the diagnosis of OI, prompt treatment with bisphosphonate was introduced as soon as the IO was genetically confirmed.

Although DXA is the most commonly used method to assess BMD and represents the gold standard in all age groups (7), this technique has several limitations in the newborn period. Besides its limited availability and the need for neonatal transport, a matter of concern is also the cumulative radiation dosage associated with repeated measurements (3). The variables provided by DXA (bone mineral content and BMD) are significantly confounded by changes in the size and shape of the skeleton, as well as in the amount of soft tissue occurring during the rapid growth of neonates (5, 8). Because DXA measurements are affected by bone size, they are less reliable in children below the age of 4 years (3) and in children suffering from abnormal growth patterns, such as being small for their age, and in subjects with delayed sexual maturation. Since DXA results depend on a two-dimensional projection of a three-dimensional structure, true volumetric BMD is not obtained (3). In addition, there is a lack of reference data for children under two years of age (1).

In contrast, QUS assesses connectivity and elasticity, and thus provides a measure of “bone quality”. The reproducibility is high, the devices are portable, and measurements are quickly feasible. QUS gives information about bone micro-architecture in addition to BMD, by measuring the time taken by the ultrasound signal to travel through the bone tissue. These propagation times between the two transmitters and two receivers contained within the QUS probe are used by a proprietary algorithm to determine the bone speed of sound (SOS), which is expressed as meters per second (m/s) (9) and is a pure parameter of velocity independent of ultrasound wave attenuation. Other variables for assessing BMD are amplitude-dependent SOS (AD-SOS) and bone transmission time (BTT), which are usually measured by the phalangeal QUS device. SOS is a variable usually measured by QUS methods applied to the heel, radius, tibia, and patella (10). In our cases, BMD was measured by SOS on the cortical bone at the right tibia using the QUS Sunlight Omnisense 7000P (Sunlight Medical Ltd, Tel Aviv, Israel). The software uses the three most consistent measurements to compute the result. Bone measurements are expressed in Z-scores for age, height and pubertal stage, according to the QUS device used. Just as in the interpretation of DXA in children, a measurement below −2 SD identifies bone health impairment, or “low bone mineral status” in relation to the anthropometric variable considered (9).

As QUS and DXA assess different bone tissue properties, occasional divergent results between them do not necessarily indicate methodological error [7], but emphasize the complementarity of the two methods (9).

However, utilization of QUS method involves some controversies. Some researchers have suggested that bone size and site of measurement may also affect the accuracy of BMD measurement, especially in growing children. Another concern regarding reliability is the single BMD measuring site, which may not reflect other parts of the skeleton (3). Additionally, reference values for normal BMD are scarce in the pediatric population, especially for neonates, and may vary between ethnic groups (11–14).

Having these limitations in mind, our case series shows that this modality has potential as an initial screening tool for identification of newborns with MBD, as well as for assessing the subsequent development of bone health, including in monitoring bisphosphonate treatment response.

Knowledge of the advantages of using QUS may avoid diagnostic delays in sick newborns.

## Author contributions

SC, AŠ and DP: contributed to the conception and design of the manuscript. SC and DP: wrote the initial version of the manuscript. SC, AŠ and DP: wrote sections of the manuscript. DP and AŠ: supervised the manuscript. All authors reviewed, edited, and approved the final manuscript. All the authors have accepted responsibility for the entire content of this submitted manuscript and approved submission. All authors contributed to the article and approved the submitted version.
